# Comprehensive Forecasting of Electrical Quantities in an Educational Building via Artificial Intelligence-Driven Distributed Measurement System

**DOI:** 10.3390/s25082456

**Published:** 2025-04-14

**Authors:** Virginia Negri, Roberto Tinarelli, Lorenzo Peretto, Alessandro Mingotti

**Affiliations:** Department of Electrical, Electronic and Information Engineering, Guglielmo Marconi Alma Mater Studiorum, University of Bologna, Viale del Risorgimento 2, 40136 Bologna, Italy; virginia.negri2@unibo.it (V.N.); roberto.tinarelli3@unibo.it (R.T.); lorenzo.peretto@unibo.it (L.P.)

**Keywords:** distributed measurement system, forecasting, accuracy, artificial intelligence, digital twin, energy meters, sensors

## Abstract

Recent environmental concerns have heightened attention toward new solutions across all fields to mitigate human impact. The power system community is also deeply committed to addressing this issue, with research increasingly focused on sustainable practices. For instance, there is a growing trend in designing new buildings to be net-zero emitters, while older structures are being retrofitted for energy efficiency to achieve similar goals. To this purpose, the study aims to enhance the energy management capabilities of an educational building by implementing a smart infrastructure. Equipped with photovoltaic panels and a distributed measurement system, the building captures voltage and current data and calculates power. These electrical quantities are then forecasted through an AI-driven framework that manages the data. The paper details the AI model used, including its experimental validation. The results show that the system provides reliable forecasts of electrical parameters. The evaluation of the distributed measurement system and the collected data offers valuable insights, which support more informed actions for optimizing energy management and system performance. A key novelty of this study lies in the exploration of model generalization across measurement nodes. This approach is supported by the correlation analysis of data, which highlights the potential for accurate predictions in case of data gaps. Moreover, the ease of deployment and the practical application of the system were highlighted as key factors for scalability, allowing for potential adaptation in similar infrastructures.

## 1. Introduction

In the era of advancing technology, the integration of artificial intelligence (AI) and distributed measurement systems (DMSs) has emerged as a groundbreaking approach for developing smart and efficient infrastructures. This convergence holds significant potential, especially in the construction industry, where optimizing resources and enhancing sustainability are key objectives. One of the most critical aspects of modern power systems is the forecasting of electrical quantities, which plays a vital role in improving energy management, reducing operational costs, and enhancing grid stability [1].

Forecasting techniques are typically categorized based on time horizon: very short-term (minutes to hours), short-term (hours to days), now-casting (real-time prediction), and long-term (months to years). Among these, short-term load forecasting is the most widely applied approach in energy consumption prediction [2]. Traditional methods such as Autoregressive Integrated Moving Average (ARIMA) and Autoregressive Moving Average with Exogenous Inputs (ARMAX) have been commonly used for energy forecasting [3]. These time series methods rely on historical data and often struggle to capture nonlinear relationships [4].

More recently, machine learning (ML) and deep learning (DL) techniques, such as Long Short-Term Memory (LSTM) networks, Gated Recurrent Units (GRUs), and transformer-based models, have shown superior performance in uncovering complex patterns in energy consumption data [5]. Moreover, ensemble learning methods, including Random Forest (RF) and Gradient Boosting techniques like eXtreme Gradient Boosting (XGBoost) and Light Gradient Boosting Machine (LightGBM), have demonstrated robust predictive capabilities by leveraging multiple weak learners to improve accuracy [6]. Support Vector Machines (SVMs), known for their effectiveness in handling high-dimensional spaces and nonlinear relationships through kernel functions, have also been successfully applied in energy forecasting, offering competitive performance in certain scenarios [7].

Additionally, Convolutional Neural Networks (CNNs), originally designed for image processing, have been increasingly applied in energy forecasting due to their ability to automatically extract spatial and temporal features from raw time series data. By leveraging convolutional layers to detect local dependencies and hierarchical patterns, CNNs can enhance forecasting accuracy [8].

In this context, Digital Twins (DTs) play a crucial role in energy management by offering a real-time digital replica of physical assets [9,10]. DTs are powerful tools for predictive maintenance, enhancing sustainability, increasing efficiency, and supporting informed decision making [11]. By accurately forecasting energy consumption and detecting anomalies, DTs can predict maintenance needs, helping to prevent equipment failures and minimize downtime [12].

The effectiveness of DTs depends heavily on the forecasting models used to represent system behavior. Integrating AI within DT frameworks significantly enhances their predictive capabilities, enabling facility managers to optimize energy distribution, detect anomalies, and anticipate potential failures [13]. In the realm of energy monitoring, the integration of the Internet of Things (IoT) with DL models has been shown to enable the real-time monitoring and control of energy resources across residential buildings [14]. Similarly, DTs have been demonstrated to improve energy efficiency and occupant comfort in public and commercial buildings by replacing rule-based heating and ventilation systems with model predictive control [15].

Additionally, the accuracy of collected data plays a crucial role. Since instrument transformers (ITs) are the primary sources for voltage and current measurements in acquisition systems, their selection is critical and must align with the target uncertainty requirements of the final application. Achieving reliable data collection also requires addressing the non-ideal characteristics of signals, which can be influenced by environmental factors such as temperature and humidity. Consequently, as demonstrated in the literature, techniques for IT modeling, calibration, and compensation are essential to mitigate these influences and ensure measurement accuracy [16,17]. Of course, the contribution to the uncertainty due to the involved algorithms must be considered as well. However, it is well known that such a contribution is typically negligible, or at least one or more orders of magnitude lower than those of ITs [18].

Compared to the current literature, as noted in two general reviews [19,20] and a review on data-driven techniques [21], this paper presents a comprehensive solution integrating features that are often addressed individually.

Building on the results from [22], this paper focuses on an AI-driven forecasting framework for electrical quantities in an educational building using a DMS to collect data on voltage, current, and power consumption. The proposed framework employs an LSTM network to enhance forecasting accuracy and reliability across multiple nodes within the building. Extensive experimental testing has been conducted to fine-tune the model, focusing on data quality assessment and correlation analysis. A key feature of this research is the exploration of model generalization within the DMS. Specifically, models trained on data from one node are tested on data from another node, allowing the system to compensate for missing data at a node by predicting values from other locations, thus supporting scalability across the building.

In addition to forecasting, the study evaluates the practical limitations and implementation challenges of integrating AI into distributed energy systems to support the future development of a DT for the building. The insights from this work lay the foundation for DT-based optimization strategies, enabling predictive maintenance, enhanced energy efficiency, and adaptive decision making.

This paper presents a comprehensive solution for AI-driven energy forecasting, addressing key challenges in the field:

- Development of an AI-based forecasting model leveraging the LSTM network optimized for electrical quantities prediction.
- Evaluation of model performance across the DMS, ensuring reliable and scalable monitoring across different electrical nodes.
- Exploration of model generalization within the DMS to compensate for missing data at nodes by testing models trained on data from other nodes.
- Identification of practical implementation challenges, including sensor installation, AI model optimization, and real-world data acquisition constraints.
- Exploration of the trade-offs between forecasting accuracy, computational complexity, and ease of deployment, making the approach adaptable to different infrastructures.

Taken singularly, the contribution might not be considered a big step forward in research. However, their integration and exploitation are the key added values that provide useful insights to the scientific community.

The paper’s structure is as follows: Section 2 gives an overview of the concepts needed to enhance the comprehension of the work and the related state of the art. Section 3 summarizes the use case and provides descriptions of the DMS and the LSTM network. Section 4 details the evaluation approach. Subsequently, Section 5 presents the results and their discussion. Finally, Section 6 concludes the work.

## 2. Background

The DT concept, though relatively new, is rapidly gaining traction across sectors. Defined in IEC 30173 [23] as a “digital representation of a target entity with data connections enabling convergence between physical and digital states at a synchronized rate,” DTs are increasingly applied beyond their origins in manufacturing and aerospace to sectors like power systems [24,25]. While complete power grid digitalization is challenging, progress is underway, as demonstrated by this study, which aims to move towards the energy DT by forecasting power consumption and managing renewable energy sources.

Time series forecasting is essential in this context, with AI models showing superior performance in energy management systems [26,27]. ML and DL algorithms, such as RF, Support Vector Regression (SVR), and LSTM networks, are widely applied for energy forecasting due to their ability to capture complex data relationships. Studies like [28,29,30] showcase applications of RF, SVR, and DL models in predicting electricity demand across different intervals. Additionally, ref. [31] proposes a CNN for the electricity consumption estimation of a building in Canada.

Hybrid approaches that combine statistical methods with ML are also emerging, leveraging the strengths of both for enhanced forecasting accuracy. Traditional statistical models, such as ARIMA and ARMAX, are well suited for capturing long-term dependencies and seasonality in time series data. For instance, ARIMA-LSTM models have been widely used to exploit ARIMA’s ability to handle linear trends while allowing LSTM to capture complex nonlinear dependencies [32]. Similarly, in [33], ARMAX is combined with Neural Network (NN) to enhance predictive accuracy. Other hybrid approaches, such as Wavelet Transform–ARIMA (WT-ARIMA), further refine forecasting capabilities by decomposing time series data into different frequency components before applying statistical models [34]. These methods offer improved generalization, better handling of missing data, and reduced overfitting, making them highly effective for energy forecasting applications.

Despite the variety of techniques available, this study opts for the use of LSTM networks. LSTM has become a widely accepted method in the field, with a strong track record of success in energy forecasting, ensuring its reliability for this study. With this regard, note that the primary emphasis of this work is not on the forecasting method itself but on the development and evaluation of a comprehensive framework. The proposed approach lays the groundwork for DT technology while also addressing the analysis of data from the DMS and ensuring the model’s transferability across different nodes.

## 3. Materials and Methods

### 3.1. Overview of the Building

The framework is developed for short-term electrical consumption forecasting at the “Bertalia-Lazzaretto” Engineering Faculty of the University of Bologna, a 230,000 m^2^ complex hosting classrooms, offices, and labs. A DMS, supported by the iOtto interface from Onit [35], gathers data from diverse spaces and equipment, making this an ideal setting for AI-driven forecasting. With 440 kW of rooftop photovoltaic panels, integrating consumption forecasts into a DT framework enables matching energy use with solar production, optimizing energy efficiency and sustainability.

### 3.2. The Distributed Measurement System and Data Collection

**Figure 1:**
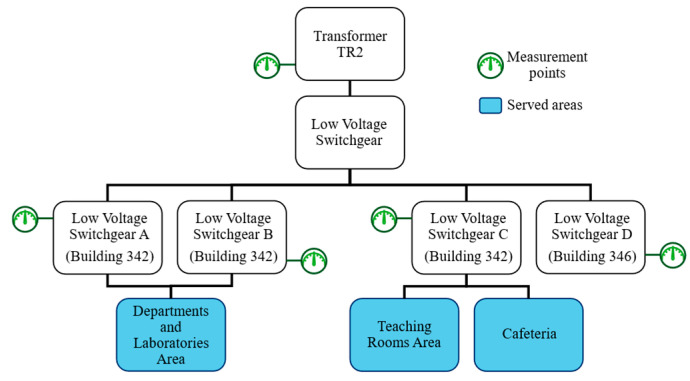
Diagram of the DMS reading points and served areas.

The deployed DMS facilitates the acquisition of diverse electrical parameters at various locations throughout the building. The block diagram presented in Figure 1 describes the structure of measurement nodes and sub-nodes. Specifically, transformer TR2 serves as the power source for two structures (Buildings 342 and 346) within the complex. Low Voltage (LV) switchboards A and B distribute power to the departments (including offices and meeting rooms) and laboratories. Switchboard C provides electricity to the lecture rooms and the building’s cafeteria, which have appliances such as ovens and refrigerators. The green symbol indicates the measurement points, while the served areas are depicted in blue.

**Figure 2:**
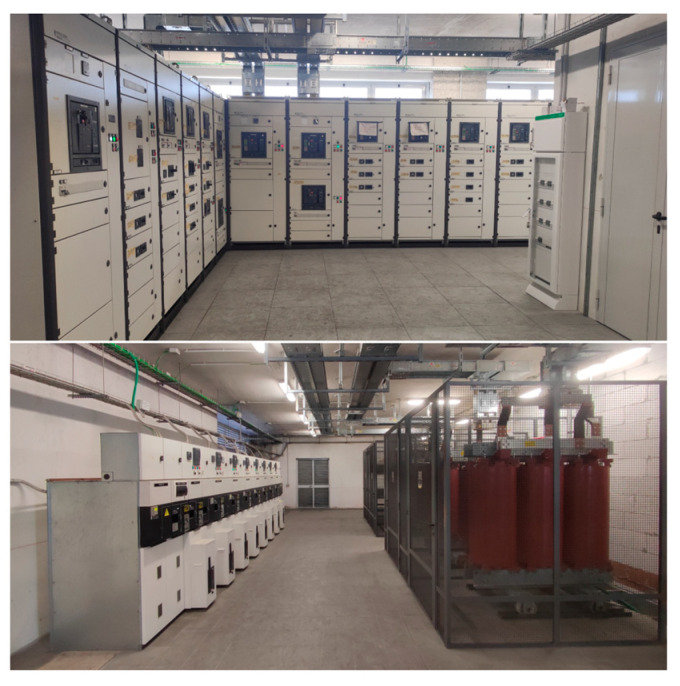
Picture from the LV and MV substations in the building.

At each point, voltage, current, active and reactive power, current and voltage Total Harmonic Distortion (THD) coefficient, and power factor measurements are acquired in every phase. The frequency of the system is also measured. The measurement instrumentation includes an EM2389 energy meter and DM3TA series Current Transformers (CTs). The meter has an accuracy of 1% on active power measurements. The installed CTs belong to class 0.5. The LV and the Medium Voltage (MV) substations are pictured in Figure 2. Considering the installed equipment, any algorithm or method to be developed must consider the constraints in terms of resolution. The acquired data are collected every 15 min throughout the day. Data have been stored since the 6 June 2023.

### 3.3. Long Short-Term Memory Model

The LSTM model is an artificial (recurrent) NN that supports bidirectional data flow (from input to output and vice versa), designed to capture long-term dependencies in sequential data. LSTM networks have memory cells and gating mechanisms (forget, input, and output gates) that control information flow, allowing them to retain or discard data selectively, making them ideal for time series modeling [36].

**Figure 3:**
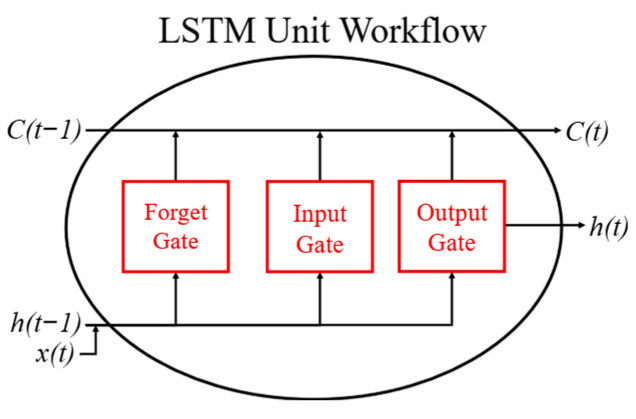
Workflow of an LSTM unit.

Figure 3 depicts a general scheme of an LSTM cell, the core of an LSTM network. C(t − 1) and C(t) represent the previous and current cell states, while h(t − 1) and h(t) are the previous and current hidden states, and x(t) is the current input [37]. The gates (forget, input, and output) use Sigmoid and Hyperbolic Tangent (Tanh) activation functions to manage information flow. The Sigmoid function limits outputs between 0 and 1, indicating how much information to retain or discard, while Tanh constrains values between −1 and 1, preventing values from becoming excessively large or small [38]. The model has been implemented using the PyTorch library (version 2.4.0) in Python programming language (version 3.12.4).

The LSTM architecture consists of two LSTM layers, which help capture more complex temporal dependencies. The hidden size was set to 64 units, providing a good balance between learning capacity and computational efficiency. The model was optimized using the Adam algorithm with a learning rate of 0.001, chosen to ensure stable convergence during training. The model was trained for 50 epochs with a batch size of 256, which allows for efficient parallel processing and reduced training time, and the loss function used was the Mean Squared Error (MSE).

### 3.4. Data Preprocessing

The data gathered from the DMS described in the previous section was used to train and test the LSTM network. This dataset includes acquisition timestamps along with values for active power, current, and voltage.

To optimize the model’s performance, input data underwent preprocessing. Initially, the timestamp feature was subjected to circular encoding, a technique specifically designed for handling time features in datasets. Circular encoding represents cyclical variables within a continuous circular space by mapping them into a circle using trigonometric functions such as sine and cosine. For instance, when encoding the time of day, each hour is projected in a unit circle, where the sine and cosine values represent the angular position of the hour hand. Subsequently, any missing data were replaced with interpolated values. Additionally, all the readings were 0 to 1 normalized.

Finally, the training and testing datasets were constructed, with the last week of each month reserved for testing and the remaining weeks allocated for training. After this split, a sliding window approach was applied to segment the data into input–output sequences, enabling the model to learn temporal dependencies effectively. Further details on the chosen input and output sequence lengths will be provided in the next section.

### 3.5. Framework

Implementing DT concepts requires a robust, flexible infrastructure for integrating data from diverse sources (e.g., the DMS) and enabling modular extensions. Users should be able to add modules for tasks like data analytics, AI-based forecasting, and visualization. The AI model needs to provide accurate energy consumption forecasts, and the DMS must offer continuous, high-resolution data acquisition. This framework achieves these aims, leveraging the LSTM network’s capacity to analyze sequential data and predict trends, while the DMS collects detailed electrical parameters from various building points.

**Figure 4:**
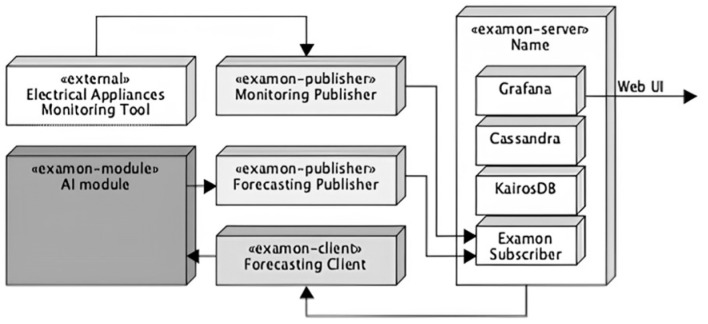
ExamonX framework.

To support these requirements, the ExamonX [39] framework was selected. Designed for IoT monitoring and predictive maintenance, ExamonX integrates components for data flow and processing, with Cassandra for database management, KairosDB for time-series support, and Grafana for visualization. ExamonX includes a software development kit for creating publisher applications, which adapt and send external data to the system. Figure 4 illustrates the ExamonX architecture, with an AI forecasting module (housing the LSTM model) and two publishers. The “monitoring publisher” inputs data from the DMS, while the “forecasting publisher” inputs data generated by the AI module. This design separates data sources from processing algorithms, ensuring scalability and modularity, and allowing easy expansion for future features. All the data and analytics are accessible through Grafana’s web interface.

## 4. Test Description

### 4.1. Performance Across DMS Points and Quantities

The performance of the LSTM network in forecasting trends of electrical quantities was evaluated at various points throughout the DMS. Specifically, TR2, LV Switchgear (LVS)-A, -B, and -D were considered as measurement points. The selection of measurement points was based on the distribution of electrical loads across the building. TR2 was chosen as a measurement point because it is the upstream transformer, providing power to the entire system. LVS-A and LVS-B were selected because they feed the same section of the building, specifically, the departments and laboratories, which include heavy machinery that can significantly influence energy consumption patterns. Lastly, LVS-D was included, as it feeds a separate building, allowing for an analysis of energy consumption in a different part of the facility.

As described in Section 3, the dataset includes measurements taken every 15 min of active power, current, and voltage at the selected points, spanning from 6 June 2023 to 17 October 2024, totaling 47,485 data points for each quantity at each reading point. The choice of quantities was made to select key parameters both for forecasting energy consumption and for monitoring power quality. Specifically, the active power measurement represents the total active power across all three phases, as it is essential to consider the overall power consumption of the building rather than focusing on individual phase values. The current measurement is taken from phase one as it provides a representative indication of the electrical load in that phase. The voltage is measured between phase one and neutral, which is critical for monitoring the system’s voltage stability.

The initial phase of testing focused on evaluating the performance of the model for different input sequence lengths and forecast horizons at the TR2 measurement point. The objective was to determine how variations in input sequence length affected model performance. Once this parameter was optimized for TR2, the results were extended to the other measurement points.

### 4.2. Data Correlation Analysis

To better understand the relationships between the predicted electrical quantities, the Pearson correlation has been computed among these variables for each considered reading point. Correlation analysis is valuable in revealing how changes in one variable might be associated with changes in others, providing insights into the system’s dynamics and dependencies.

The Pearson correlation coefficient *r* is a statistical measure that quantifies the strength and direction of the linear relationship between two variables [40]. The coefficient ranges from −1 to 1, where

- *r* = 1: Perfect positive linear correlation (as one variable increases, the other does as well).
- *r* = 0: No linear correlation.
- *r* = −1: Perfect negative linear correlation (as one variable increases, the other decreases).

The Pearson correlation coefficients were calculated pairwise for power, current, voltage, and frequency at all the considered measurement points (TR2, LVS-A, LVS-B, and LVS-D).

### 4.3. Model Transferability: Testing on Correlated Points

To comprehensively evaluate the predictive model and its potential within the considered DMS, an additional assessment was conducted. Given the correlation between the variables measured at different points in the system, the possibility of leveraging data from one point to predict measurements at another was explored. Initially, the LSTM network was trained using data from the LVS-A measurement point. Subsequently, the trained model was used to forecast the electrical quantities at a different measurement point, specifically LVS-B. This step was designed to assess the model’s transferability and its ability to predict trends across various locations within the system, even when trained on data from a distinct reading point. The objective was to determine how well the model could be extended to other parts of the DMS, particularly for locations with limited or no historical data, and to evaluate its generalizability across the system.

### 4.4. Comparison with State-of-the-Art Methods

Finally, the LSTM model results were compared with those obtained from the widely used ML and traditional methods, specifically, RF, ARIMA, and CNN. The comparison was made based on the same test cases as the initial LSTM model evaluation. For the sake of brevity, only the TR2 measurement point acquisitions (power, current, and voltage) were used.

The ARIMA model was implemented using the statsmodels Python library (version 0.14.4). The order of the ARIMA model was set to (*p* = 5, *d* = 1, and *q* = 0) for the autoregressive, differencing, and moving average components, respectively. The seasonal component was initially considered in the model but was later omitted due to its significant impact on training times.

The Random Forest model was implemented using the scikit-learn Python library (version 1.6.0). The model was configured with 100 estimators (trees).

For the CNN model, a custom architecture was defined using PyTorch (version 2.4.0). The model consisted of two 1D convolutional layers, with 32 and 64 output channels, respectively, and kernel sizes of 3. These were followed by max-pooling layers to down-sample the feature maps. After the convolutional layers, the output was flattened and passed through two fully connected layers, with the final layer outputting the predicted values. The number of hidden units in the fully connected layers was set to 64. The model used Rectified Linear Unit (ReLU) activation after each convolutional and fully connected layer, and the Adam optimizer was used for training with a learning rate of 0.001. The model was trained for 50 epochs with a batch size of 256, and the loss function used was MSE, as for the LSTM model.

### 4.5. Evaluation Metrics

Various metrics are used to evaluate model performance in energy consumption forecasting, including Mean Absolute Error (*MAE*), Mean Absolute Percentage Error (*MAPE*), Root Mean Square Error (*RMSE*), and the Coefficient of Determination (*R*^2^) [41].

*MAE* is defined as follows:

(1) $$MAE=\frac{1}{n}\sum_{i=1}^{n}\left|y_{i}-\hat{y_{i}}\right|,$$

where n is the total number of estimations, $y_{i}$ the reference value, and $\hat{y_{i}}$ the estimated value. *MAE* metric measures the average of the absolute differences between predictions and actual values.

*RMSE* is defined as follows:

(2) $$RMSE=\sqrt{\frac{1}{n}\sum_{i=1}^{n}{\left(y_{i}-\hat{y_{i}}\right)}^{2}},$$

where quantities have been already defined. *RMSE* calculates the square root of the average of the squared differences between predictions and actual values, offering a measure of the standard deviation of errors.

*MAPE*, defined as

(3) $$MAPE=\frac{1}{n}\sum_{i=1}^{n}\left|\frac{y_{i}-\hat{y_{i}}}{y_{i}}\right|,$$

assesses the accuracy of predictions by averaging the absolute percentage errors relative to actual values.

Lastly, the *R*^2^ score is defined as follows:

(4) $$R^{2}=1-\frac{\sum_{i=1}^{n}{\left(y_{i}-\hat{y_{i}}\right)}^{2}}{\sum_{i=1}^{n}{\left(y_{i}-\bar{y}\right)}^{2}},$$

where $\bar{y}$ is the mean of the actual values. The *R*^2^ score indicates the proportion of variance in the dependent variable (output feature) explained by the independent variables (input features) of the model. *R*^2^ scores range from 0 to 1, with higher values indicating better predictive ability. Four different metrics were selected to exploit their different meaning to have a comprehensive evaluation of the forecasting approach.

## 5. Test Results and Discussion

### 5.1. Performance Across DMS Points and Quantities

Table 1 provides the metric error values for different input sequence lengths and forecast horizons for the forecasting of active power at the TR2 point. The input sequence length includes 4, 8, 16, and 96 data points, respectively, equal to the preceding 1, 2, 4, and 24 h. The forecasting horizon includes 1, 4, 16, and 96 output values, representing intervals of 15 min, 1, 4, and 24 h into the future, respectively. The results indicate that an 8-point sequence, corresponding to the previous 2 h, strikes a good balance between low errors (i.e., high performance) and minimal input data requirements. These results were extended to all the quantities and measurement points, with a 2 h input sequence selected for all the cases. Table 2, Table 3, Table 4 and Table 5 present the metrics obtained for each quantity across different forecast horizons for TR2, LVS-A, -B, and -D, respectively.

The results demonstrate the LSTM model’s accuracy and adaptability across various time horizons, electrical quantities, and measurement points. Shorter time horizons, such as 15 min, consistently yield lower errors and higher *R*^2^ values, indicating stronger predictive accuracy. As the forecast horizon extends, a slight increase in error metrics and a decrease in *R*^2^ values is observed, reflecting the typical trade-off between forecast accuracy and horizon length. In general, the model achieves the best results at TR2 and LVS-A points, where the *MAPE* and *R*^2^ values indicate high predictive accuracy across both short and extended forecast horizons.

Overall, the LSTM model provides robust forecasting for different electrical quantities, with optimal performance at shorter horizons. A comment on the *MAPE*—in some cases, the presented results are zero. This is due to the extremely low error values and the choice of significant digits used to represent it.

**Figure 5:**
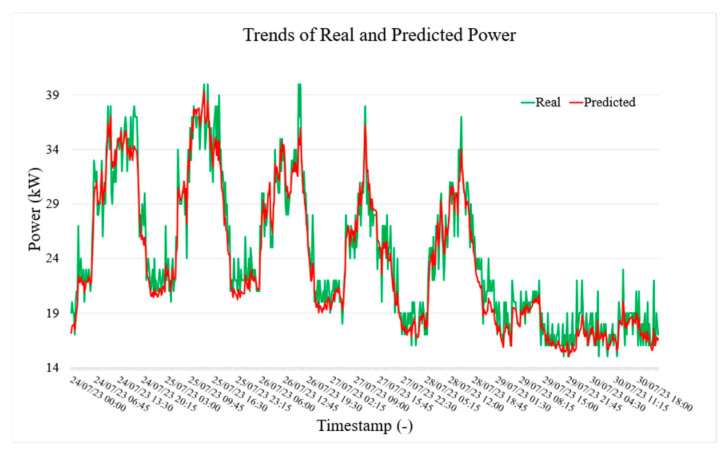
Trend of real and predicted power test values of LVS-A measurement point.

**Figure 6:**
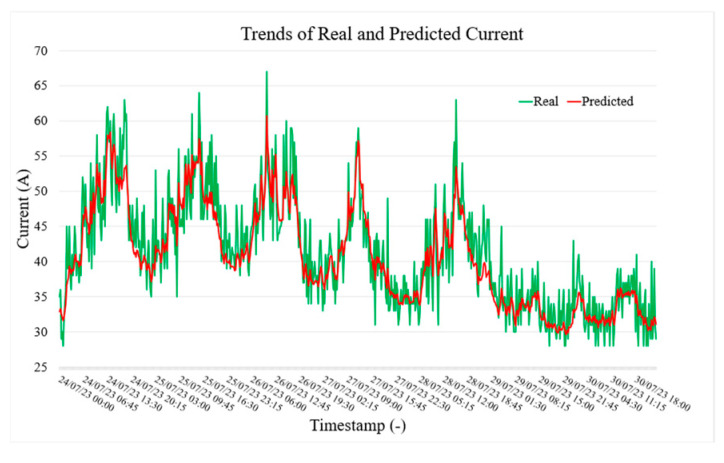
Trend of real and predicted current test values of LVS-A measurement point.

**Figure 7:**
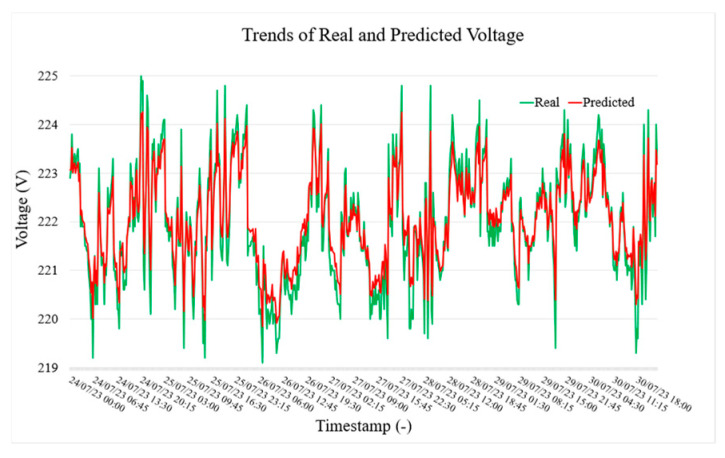
Trend of real and predicted voltage test values of LVS-A measurement point.

Figure 5, Figure 6 and Figure 7, respectively, show the actual (green) and predicted (red) power, current, and voltage values in LVS-A over a week from 24 July to 30 July. The model forecasts current values 15 min ahead using data from the previous 2 h. In general, the predicted trend closely follows the actual values, capturing general patterns and trends. However, some differences are evident during rapid fluctuations, where the predicted line smooths out sharp peaks (but still within the accuracy limits given by the specification). Despite this, the visualization demonstrates satisfactory predictive accuracy, though some refinement may be needed for extreme variations (outside the scope of the paper).

### 5.2. Data Correlation Analysis

**Figure 8:**
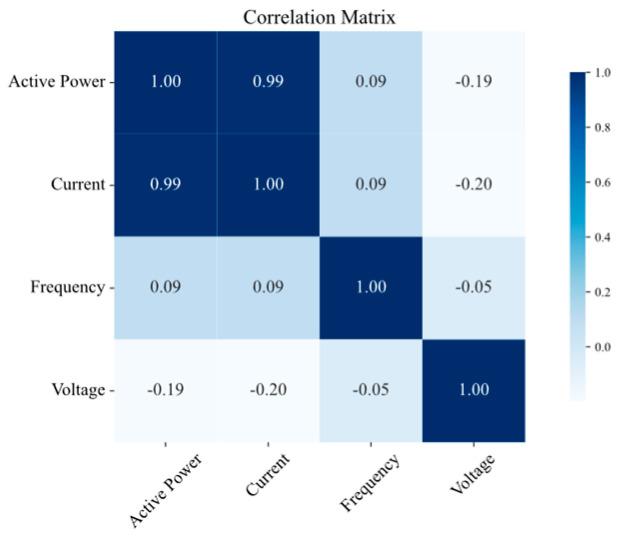
Correlation matrix of electrical quantities on TR2 measurement point.

**Figure 9:**
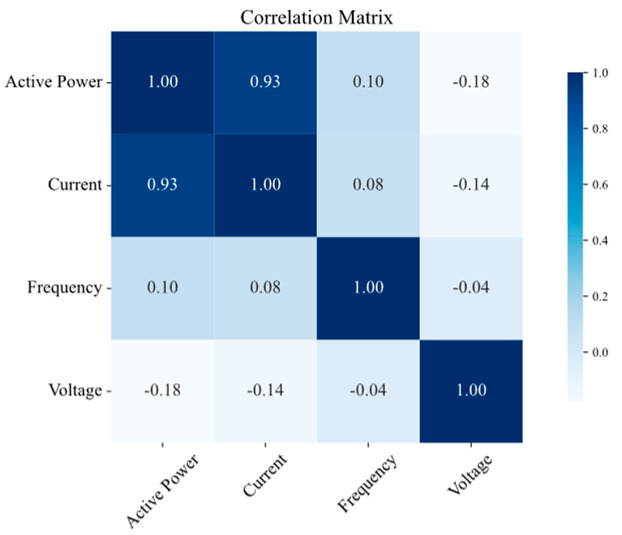
Correlation matrix of electrical quantities on LVS-A measurement point.

**Figure 10:**
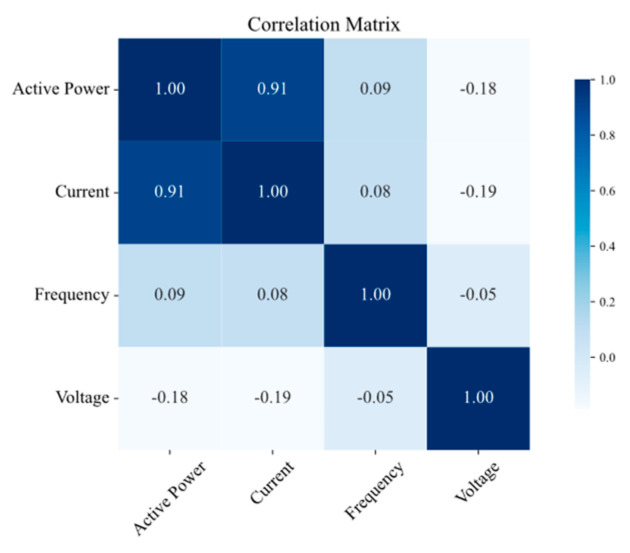
Correlation matrix of electrical quantities on LVS-B measurement point.

**Figure 11:**
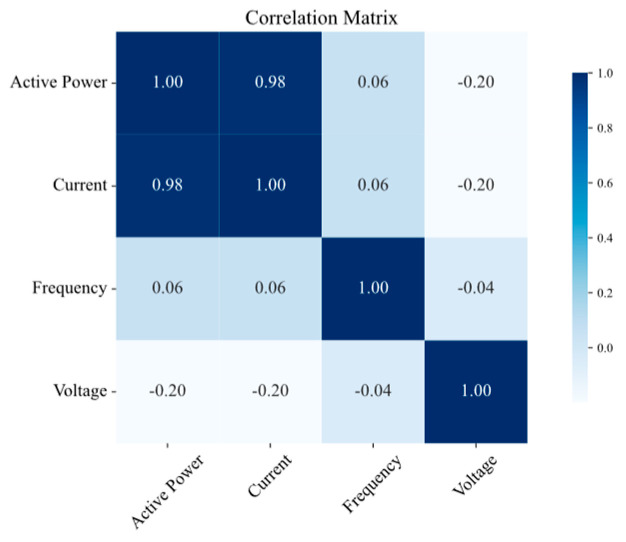
Correlation matrix of electrical quantities on LVS-D measurement point.

The results of the correlation analysis are summarized in Figure 8, Figure 9, Figure 10 and Figure 11 for the measurement points TR2, LVS-A, -B, and -D, respectively. Active power consistently shows a strong positive correlation with the current across all the measurement points, which is expected, as an increase in active power is generally associated with an increase in current in electrical systems. Conversely, frequency exhibits a low correlation with both power and current, aligning with the fact that frequency is typically regulated and unaffected by load fluctuations in well-managed electrical systems. Voltage shows a slightly negative correlation with both power and current, suggesting a subtle compensatory behavior, where increases in load (and thus power and current) might lead to slight decreases in voltage.

**Figure 12:**
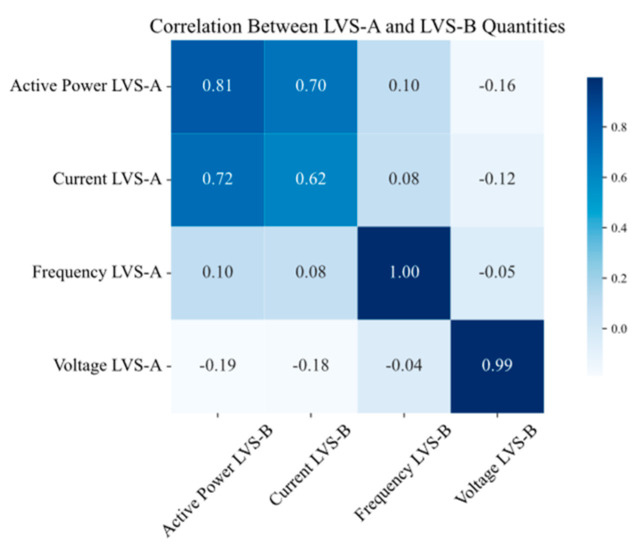
Correlation matrix of electrical quantities on LVS-A and LVS-B measurement points.

To further explore the DMS, the correlation between variables from two different measurement points, LVS-A and LVS-B, was also investigated to understand how the electrical quantities are related across different points. Figure 12 represents the correlation values between the two measurement points. The results indicate a high correlation between the same quantities, suggesting that data from one point could potentially be used to infer information from the other. This analysis is particularly valuable because there could be missing data in one or more nodes for various reasons. By identifying relationships between quantities and across specific nodes, the forecasting method can provide more accurate results, even under abnormal conditions where data might be missing.

### 5.3. Model Transferability: Testing on Correlated Point

Table 6 presents the resulting error metrics for the LVS-B measurement point, achieved with the model trained on LVS-A data and considering eight-input acquisitions and 1, 4, 16, and 96 output points, equivalent to a 15 min, 1, 4, and 24 h projection into the future.

The results can be explained by the correlation between the variables at LVS-A and LVS-B. The model performed best for voltage, where the correlation was almost perfect between the two points, making it easier to predict these quantities accurately. In contrast, power and current showed weaker correlations, which led to decreased accuracy, particularly for longer forecasting horizons. Also, in this case, the model performs well for shorter forecasting intervals, with low values of *MAE* and *RMSE*, especially considering the range of variation in the quantities. Overall, the performance is still good and acceptable, demonstrating the model’s transferability. The results suggest that the model can effectively predict quantities across different points in the DMS, demonstrating the model’s robustness and adaptability.

### 5.4. Comparison with State-of-the-Art Methods

Table 7, Table 8 and Table 9 present the resulting error metrics for the power forecasting of the TR2 measurement point, achieved with the RF, ARIMA, and CNN methods, respectively. An input sequence of eight acquisitions and 1, 4, 16, and 96 output points, equivalent to a 15 min, 1, 4, and 24 h projection into the future, have been used. The CNN model consistently outperforms the other methods, achieving the lowest error metrics and highest *R*^2^ values, particularly for shorter time horizons. The RF model performs well, especially for the 15 min and 1 h forecasts, while ARIMA shows significantly poorer performance, especially for longer time horizons.

**Figure 13:**
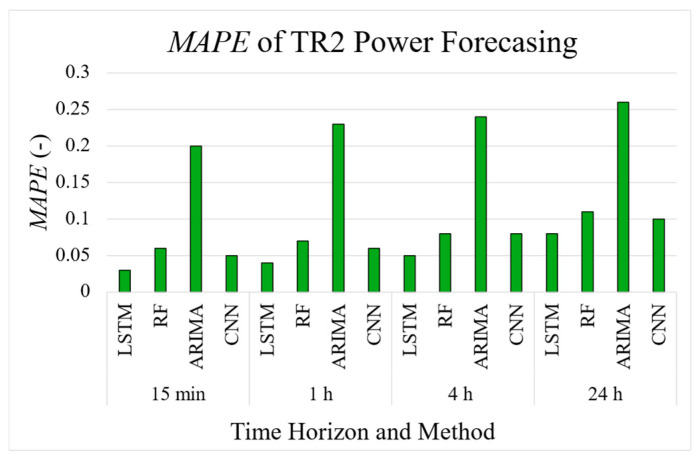
Comparison of *MAPE* metric values for power forecasting on TR2 measurement point for all the considered methods.

To compare the LSTM model with the results from the other methods, Figure 13 displays the *MAPE* values obtained for power forecasting at the TR2 measurement point using an input sequence of eight data points (2 h) and various time horizons. The results demonstrate that the LSTM model consistently achieves lower *MAPE* values across all the time horizons.

Finally, Table 10 presents the training and testing times for power forecasting at the TR2 measurement point with a 2 h input sequence and 4 h time horizon. The results show that the ARIMA method has the shortest training and testing times, making it computationally efficient, although its forecasting performance is very poor. In comparison, the LSTM and CNN models require significantly more time for training. However, both LSTM and CNN offer superior forecasting performance.

### 5.5. Final Discussion

#### 5.5.1. Target Uncertainty

The evaluation of the results should also account for the target uncertainty of the application. The absolute indices presented in Subsection B are satisfactory when considering the uncertainty associated with the equipment used. Specifically, the 1% accuracy for power and the 0.5 accuracy class of the CT are appropriate for the application’s requirements and desired forecasting precision. Consequently, if smaller variations in quantities are required, the equipment should be chosen to match that need. Therefore, as demonstrated in this project, when applying the established uncertainty propagation rules, the entire measurement chain used to obtain key quantities and parameters must be carefully verified to calculate the overall uncertainty.

#### 5.5.2. Challenges of Digital Twin Implementation

The promising results from short-term active power forecasting in the DMS suggest that integrating the AI model is crucial for developing the building’s DT. This is particularly true considering the challenging requirements for building an accurate DT. In fact, AI could avoid the need for accurate models and deep knowledge of the involved assets. Using a single LSTM model to forecast multiple measurement points could optimize implementation. This approach may be extended to not only consumption forecasting but also energy quality assessment, anomaly detection, and system health monitoring.

For the energetic DT, additional data like schedules, holidays, and room occupancy (possibly via CO_2_ sensors) are needed, increasing system complexity. A compelling research direction involves energy redistribution with electric vehicles (vehicle-to-grid), using DT data to manage charging/discharging based on photovoltaic production. This scenario also introduces the need for long-term forecasting, presenting new possibilities for strategic energy planning.

## 6. Conclusions

This study presents a robust AI-based solution for enhancing energy management in educational buildings through a distributed measurement system. By forecasting critical electrical quantities—such as voltage, current, and power—the approach provides real-time insights that support efficient energy use, renewable integration, and operational cost reduction. The system’s capacity to forecast even in the presence of missing data demonstrates its resilience and utility in dynamic environments. The paper highlights the importance of reliable data collection, addressing the uncertainties related to instrument transformers and environmental influences, which are vital for maintaining measurement accuracy. Through extensive experimental testing and validation, the model proves effective in optimizing the performance of distributed measurement systems. Overall, the integration of AI in this infrastructure offers a promising foundation for the development of DT, enhancing energy management, predictive maintenance, and operational insights. By combining diverse technological elements—AI, distributed sensing, data management, and cloud systems—into a cohesive framework, this work contributes a comprehensive approach to sustainable energy management in complex infrastructures.

## Figures and Tables

**Table 1:** LSTM metrics with different input sequence lengths and time horizons of power on TR2 measurement point.

Input Sequence	Time Horizon	*MAE* (kW)	*RMSE* (kW)	*MAPE* (-)	*R*^2^ (-)
1 h	15 min	4.06	5.24	0.04	0.98
1 h	1 h	6.19	8.28	0.05	0.94
1 h	4 h	8.26	11.33	0.07	0.88
1 h	24 h	11.60	17.37	0.10	0.73
2 h	15 min	4.50	5.77	0.03	0.99
2 h	1 h	6.45	8.50	0.04	0.93
2 h	4 h	8.03	11.11	0.05	0.90
2 h	24 h	11.67	17.19	0.08	0.76
4 h	15 min	4.83	6.13	0.04	0.97
4 h	1 h	6.41	8.44	0.06	0.94
4 h	4 h	7.96	10.94	0.07	0.89
4 h	24 h	11.64	17.23	0.10	0.73
24 h	15 min	4.60	5.96	0.04	0.97
24 h	1 h	6.30	8.29	0.06	0.94
24 h	4 h	7.84	10.72	0.07	0.90
24 h	24 h	12.21	20.12	0.11	0.64

**Table 2:** LSTM metrics with different time horizons of electrical quantities on TR2 measurement point.

Electrical Quantity	Time horizon	*MAE*	*RMSE*	*MAPE* (-)	*R*^2^ (-)
Current	15 min	7.89 A	10.18 A	0.05	0.96
Current	1 h	11.22 A	14.86 A	0.06	0.92
Current	4 h	13.90 A	19.15 A	0.08	0.87
Current	24 h	19.60 A	28.91 A	0.11	0.71
Voltage	15 min	0.25 V	0.35 V	0.00	0.92
Voltage	1 h	0.60 V	0.80 V	0.00	0.57
Voltage	4 h	0.81 V	1.02 V	0.00	0.40
Voltage	24 h	0.90 V	1.12 V	0.00	0.16

**Table 3:** LSTM metrics with different time horizons of electrical quantities on LVS-A measurement point.

Electrical Quantity	Time Horizon	*MAE*	*RMSE*	*MAPE* (-)	*R*^2^ (-)
Power	15 min	0.97 kW	1.29 kW	0.05	0.97
Power	1 h	1.58 kW	2.18 kW	0.08	0.91
Power	4 h	1.97 kW	2.78 kW	0.09	0.86
Power	24 h	2.47 kW	3.58 kW	0.12	0.77
Current	15 min	2.50 A	3.24 A	0.07	0.9
Current	1 h	3.31 A	4.39 A	0.09	0.82
Current	4 h	3.86 A	5.15 A	0.11	0.75
Current	24 h	4.50 A	6.26 A	0.12	0.63
Voltage	15 min	0.22 V	0.75 V	0.00	0.93
Voltage	1 h	0.62 V	0.80 V	0.00	0.57
Voltage	4 h	0.82 V	1.01 V	0.00	0.41
Voltage	24 h	0.92 V	1.14 V	0.00	0.14

**Table 4:** LSTM metrics with different time horizons of electrical quantities on LVS-B measurement point.

Electrical Quantity	Time Horizon	*MAE*	*RMSE*	*MAPE* (-)	*R*^2^ (-)
Power	15 min	0.80 kW	1.06 kW	0.06	0.96
Power	1 h	1.17 kW	1.61 kW	0.08	0.91
Power	4 h	1.48 kW	2.07 kW	0.11	0.85
Power	24 h	2.03 kW	2.92 kW	0.15	0.70
Current	15 min	2.11 A	2.89 A	0.08	0.92
Current	1 h	2.81 A	3.90 A	0.11	0.85
Current	4 h	3.58 A	4.84 A	0.14	0.77
Current	24 h	4.35 A	6.07 A	0.17	0.64
Voltage	15 min	0.21 V	0.74 V	0.00	0.92
Voltage	1 h	0.60 V	0.81 V	0.00	0.58
Voltage	4 h	0.61 V	1.03 V	0.00	0.40
Voltage	24 h	0.93 V	1.18 V	0.00	0.15

**Table 5:** LSTM metrics with different time horizons of electrical quantities on LVS-D measurement point.

Electrical Quantity	Time Horizon	*MAE*	*RMSE*	*MAPE* (-)	*R*^2^ (-)
Power	15 min	3.06 kW	4.30 kW	0.12	0.87
Power	1 h	4.16 kW	5.62 kW	0.18	0.78
Power	4 h	4.66 kW	6.47 kW	0.19	0.70
Power	24 h	5.49 kW	7.62 kW	0.23	0.59
Current	15 min	5.45 A	7.47 A	0.15	0.88
Current	1 h	7.28 A	10.24 A	0.19	0.77
Current	4 h	8.44 A	12.12 A	0.22	0.67
Current	24 h	9.96 A	14.07 A	0.27	0.56
Voltage	15 min	0.23 V	0.78 V	0.00	0.90
Voltage	1 h	0.48 V	0.79 V	0.00	0.57
Voltage	4 h	0.62 V	1.05 V	0.00	0.42
Voltage	24 h	0.95 V	1.20 V	0.00	0.16

**Table 6:** LSTM metrics with different time horizons of electrical quantities on LVS-B measurement point with LSTM model trained on LVS-A data.

Electrical Quantity	Time Horizon	*MAE*	*RMSE*	*MAPE* (-)	*R*^2^ (-)
Power	15 min	1.96 kW	2.58 kW	0.12	0.76
Power	1 h	2.78 kW	3.70 kW	0.15	0.60
Power	4 h	4.22 kW	5.50 kW	0.25	0.46
Power	24 h	5.13 kW	6.60 kW	0.34	0.32
Current	15 min	3.40 A	4.46 A	0.11	0.85
Current	1 h	4.08 A	5.65 A	0.17	0.59
Current	4 h	7.51 A	8.08 A	0.24	0.43
Current	24 h	8.98 A	10.09 A	0.31	0.30
Voltage	15 min	0.95 V	1.09 V	0.00	0.46
Voltage	1 h	1.15 V	1.75 V	0.01	0.38
Voltage	4 h	2.02 V	2.59 V	0.01	0.30
Voltage	24 h	2.82 V	3.01 V	0.02	0.26

**Table 7:** RF metrics with different time horizons of electrical quantities on TR2 measurement point.

Electrical Quantity	Time Horizon	*MAE*	*RMSE*	*MAPE* (-)	*R*^2^ (-)
Power	15 min	6.23	9.24	0.06	0.93
Power	1 h	7.23	10.96	0.07	0.90
Power	4 h	10.41	13.11	0.09	0.84
Power	24 h	13.85	22.12	0.11	0.66
Current	15 min	11.35 A	14.99 A	0.07	0.91
Current	1 h	12.99 A	17.40 A	0.08	0.88
Current	4 h	16.30 A	23.02 A	0.10	0.82
Current	24 h	22.23 A	32.04 A	0.14	0.62
Voltage	15 min	0.52 V	0.74 V	0.00	0.63
Voltage	1 h	0.77 V	0.99 V	0.01	0.34
Voltage	4 h	0.93 V	1.15 V	0.01	0.12
Voltage	24 h	1.05 V	1.32 V	0.01	0.12

**Table 8:** ARIMA metrics with different time horizons of electrical quantities on TR2 measurement point.

Electrical Quantity	Time Horizon	*MAE*	*RMSE*	*MAPE* (-)	*R*^2^ (-)
Power	15 min	18.50 kW	25.31 kW	0.20	0.10
Power	1 h	22.60 kW	28.18 kW	0.23	0.04
Power	4 h	25.07 kW	32.92 kW	0.24	0.00
Power	24 h	28.42 kW	35.38 kW	0.26	0.00
Current	15 min	35.20 A	49.73 A	0.23	0.10
Current	1 h	36.22 A	51.01 A	0.24	0.08
Current	4 h	40.02 A	55.05 A	0.25	0.04
Current	24 h	44.25 A	58.73 A	0.25	0.00
Voltage	15 min	1.39 V	1.69 V	0.01	0.05
Voltage	1 h	1.39 V	1.69 V	0.01	0.05
Voltage	4 h	1.39 V	1.69 V	0.01	0.05
Voltage	24 h	1.39 V	1.69 V	0.01	0.05

**Table 9:** CNN metrics with different time horizons of electrical quantities on TR2 measurement point.

Electrical Quantity	Time Horizon	*MAE*	*RMSE*	*MAPE* (-)	*R*^2^ (-)
Power	15 min	4.80 kW	6.35 kW	0.05	0.96
Power	1 h	4.33 kW	9.13 kW	0.06	0.92
Power	4 h	8.88 kW	13.01 kW	0.08	0.88
Power	24 h	13.28 kW	19.83 kW	0.1	0.73
Current	15 min	8.98 A	11.96 A	0.06	0.95
Current	1 h	13.01 A	15.93 A	0.07	0.90
Current	4 h	14.88 A	20.04 A	0.09	0.84
Current	24 h	21.20 A	30.12 A	0.13	0.70
Voltage	15 min	0.30 V	0.45 V	0.00	0.87
Voltage	1 h	0.65 V	0.84 V	0.00	0.51
Voltage	4 h	0.85 V	1.07 V	0.00	0.29
Voltage	24 h	0.98 V	1.18 V	0.00	0.15

**Table 10:** Training and testing time for power forecasting on TR2 measurement point for all the considered methods—4 h time horizon.

Method	Training Time (s)	Testing Time (s)
LSTM	37.03	0.18
RF	6.75	0.07
ARIMA	1.23	0.07
CNN	31.46	0.13

## Data Availability

Data are unavailable due to privacy reasons.

## Abbreviations

The following abbreviations are used in this manuscript:

AI	artificial intelligence
DMS	distributed measurement system
ARIMA	Autoregressive Integrated Moving Average
ARMAX	Autoregressive Moving Average with Exogenous Inputs
ML	machine learning
DL	deep learning
LSTM	Long Short-Term Memory
GRU	Gated Recurrent Unit
RF	Random Forest
XGBoost	eXtreme Gradient Boosting
LightGBM	Light Gradient Boosting Machine
SVM	Support Vector Machine
CNN	Convolutional Neural Network
DT	Digital Twin
IoT	Internet of Things
IT	instrument transformer
SVR	Support Vector Regression
NN	Neural Network
WT	Wavelet Transform
LV	Low Voltage
THD	Total Harmonic Distortion
CT	Current Transformer
LVS	Low Voltage Switchgear
MV	Medium Voltage
Tanh	Hyperbolic Tangent
MSE	Mean Squared Error
ReLU	Rectified Linear Unit
*MAE*	Mean Absolute Error
*RMSE*	Root Mean Squared Error
*MAPE*	Mean Absolute Percentage Error
